# Quantitative and qualitative analysis of Argentine breast cancer prevention campaigns disseminated by still images on social networks during October 2019

**DOI:** 10.17843/rpmesp.2022.392.11019

**Published:** 2022-06-30

**Authors:** Sofía Victoria Gürtler, Manuel Rodríguez-Tablado, Mariela Alejandra Weisbrot, María Victoria Ruiz-Yanzi, Karin Silvana Kopitowski, Sergio Adrián Terrasa

**Affiliations:** 1 Hospital Italiano de Buenos Aires, Ciudad Autónoma de Buenos Aires, Argentina. Hospital Italiano de Buenos Aires Ciudad Autónoma de Buenos Aires Argentina; 2 Universidad Nacional de Lanús, Remedios de Escalada, Buenos Aires, Argentina. Universidad Nacional de Lanús Universidad Nacional de Lanús Remedios de Escalada Buenos Aires Argentina

**Keywords:** Breast Neoplasms, Mammography, Early Detection of Cancer, Communications Media, Health Knowledge, Attitudes, Practice, Social Media

## Abstract

Objectives. To quantitatively document the degree of compliance of institutional messages broadcast on social networks with the recommendations of the National Cancer Institute (INC) in Argentina during October 2019, in the context of breast cancer prevention campaigns, and to qualitatively analyze the pictorial and textual elements that make up their dissemination pieces. Materials and methods. Quantitative and qualitative analysis of 171 dissemination pieces issued during October 2019 by 54 institutions, based on the evaluation of their compliance with INC recommendations, the description of the main discordant recommendations (quantitative analysis) and the qualitative analysis of 30 pieces. Results. None of the issued messages mentioned potential screening harms. Only the messages of the National Ministry of Health complied completely with the INC recommendations, while the remaining ones recommended mammograms at younger ages or at shorter intervals. Breast self-examination was the most frequent recommendation among those who didn’t comply. The images of female bodies linked to common stereotypes of sex and beauty, and paternalistic discourses appealing to fear and guilt were predominant. Conclusions. The messages broadcasted in the analyzed diffusion pieces did not comply with the INC recommendations, despite the fact that the latter are supported by scientific evidence. On the other hand, the messages reinforce sex and beauty stereotypes, guilt and the medical-hegemonic model.

## INTRODUCTION

The National Cancer Institute (INC) of Argentina recommends that women between 50 and 70 years of age should have a mammogram at least every two years, together with a breast examination by a health professional ^1^; and that the decision should be personalized for women between 40 and 50 years of age and for those over 70 years of age ^2^. These recommendations are consistent with those of the World Health Organization and the American and Canadian Preventive Services ^3^ ^-^ ^5^.

Breast cancer screening is associated with potential harms (false positives, overdiagnosis and overtreatment) and its benefits are not as clear as initially thought ^6^ ^-^ ^8^. It is estimated that if 2,000 women had mammograms for ten years, one death would be avoided, and overdiagnosis and/or overtreatment would occur in ten of them ^9^. Likewise, in 1980, the probability that a woman would receive a diagnosis of breast cancer during her lifetime was one in twelve and today that figure has risen to one in eight ^10^. Furthermore, since the beginning of mammography screening in the U.S., breast cancer diagnosis has increased without a similar decrease in breast cancer mortality ^11^, which could be explained by an increase in early-stage detection without a similar decrease in the incidence of advanced and/or metastatic disease. However, international research ^12^ and in Argentina ^13^ have documented that the predominant messages in the media tend to overestimate the benefits of screening and to make its potential risks invisible.

Every October in Argentina, several health institutions, scientific societies and civil society organizations issue large-scale messages on breast cancer. It has been documented that the information that circulates massively in the media can influence the opinion and decision making by the population ^(14;15)^.

However, so far there has been no systematic evaluation of the content of these campaigns in Argentina.

This study had two objectives: 1) to quantitatively document the degree of compliance of institutional messages broadcasted on social networks in Argentina during October 2019 in the context of breast cancer prevention campaigns with INC recommendations, and 2) to qualitatively analyze the iconic and textual elements of the dissemination pieces.

**KEY MESSAGES**

Motivation for the study: The topic of breast cancer is widely disseminated in mass campaigns. The National Cancer Institute (INC) recommends mammography every two years between the ages of 50 and 70 as screening. This information does not seem to be transmitted to the general population.

Main findings: Only the messages issued by the National Ministry of Health were in compliance with the INC. The promotion of breast self-examination was the most frequent among those who did not comply, and in turn gender and beauty stereotypes and the medical-hegemonic model were reinforced.

Implications: It is necessary to rethink the design of campaigns to avoid the dissemination of misinformation, and paternalistic discourses where, in addition, gender and beauty stereotypes are perpetuated.

## MATERIALS AND METHODS

### Study design

Cross-sectional study using quantitative and qualitative analysis techniques.

### Selection criteria

We selected the three open social networks with still image content with the largest number of regular users in Argentina ^16^: Facebook, Instagram and Twitter. 

Given the fragmented nature of Argentina’s health system, dissemination pieces published by representatives of the state, private and social security sectors on Facebook, Instagram and Twitter, between October 1 and 31, 2019, were identified. We included the pieces issued by the National Ministry of Health and the ministries of the 24 provinces, the 24 provincial “obras sociales”, the 11 national “obras sociales” and the 10 prepaid medicine companies with the largest number of affiliates ^17^ (Table 1). Scientific societies and civil society organizations related to breast cancer were also included.

**Table 1:** Description of the institutions whose dissemination pieces were part of the research sample.

Type of institution			Description	Chosen institutions
Health System in Argentina	Ministries of Health		Funded with resources from general revenues. Composed of the provincial and national administrative structures at ministerial level, and the network of public hospitals and health centers that provide free care to the entire population.	HM of the Nation, HM of the Autonomous City of Buenos Aires (CABA), HM of Buenos Aires, HM of Catamarca, HM of Chaco, HM of Chubut, HM of Córdoba, HM of Corrientes, HM of Entre Ríos, HM of Formosa, HM of Jujuy, HM of La Pampa, HM of La Rioja, HM of Mendoza, HM of Misiones, HM of Neuquén, HM of Rio Negro, HM of Salta, HM of San Luis, HM of San Juan, HM of Santa Cruz, HM of Santa Fe, HM of Santiago del Estero, HM of Tierra del Fuego, HM of Tucumán, HM of Tucumán.
	Compulsory Social Security Sector	Provincial	Funded by the mandatory contribution and contribution of workers and employers and integrated by the “Obras Sociales” (OS). It provides coverage to salaried workers and their families, according to branches of activity (National OS). In turn, each province has an OS that provides coverage to public employees in its jurisdiction (Provincial OSs).	OS of CABA, OS of Buenos Aires, OS of Catamarca, OS of Chaco, OS of Chubut, OS of Córdoba, OS of Corrientes, OS of Entre Ríos, OS of Formosa, OS of Jujuy, OS of La Pampa, OS of La Rioja, OS of Mendoza, OS of Misiones, OS of Neuquén, OS of Rio Negro, OS of Salta, OS of San Luis, OS of San Juan, OS of Santa Cruz, OS of Santa Fe, OS of Santiago del Estero, OS of Tierra del Fuego, OS of Tucumán.
		Nationals		Integral Medical Assistance Program (PAMI), Employees of Commerce and Civil Activities (OSECAC), Construction Personnel (OSPECON), Union of Civilian Personnel of the Nation (OSPCN), Tourism, Hotel and Gastronomy Personnel (OSUTHGRA), Oil Workers (OSPE), Union of Metallurgical Workers of the Argentine Republic (OSUOMRA), Employers’ Social Action (ASE), Argentine Health Personnel (OSPSA), “Obra social” of the Personnel of the External Control Organism (OSPOCE).
	Prepaid medicine companies		Funded by money from private individuals and/or businesses. Includes voluntary insurance entities called Prepaid Medical Companies whose services individuals and families pay for in the form of fixed monthly installments.	Organization of Direct Business Services (OSDE), Swiss Medical, Galeno, Omint, Medicus, Accord Salud, Medifé, Sancor Salud, Hospital Italiano, Hospital Británico.
Scientific societies			Associations of professionals, researchers or specialists gathered around an area of knowledge, with the objective of facilitating scientific discoveries and disseminating this knowledge.	Argentine Society of Mastology, Argentine Federation of Gynecology and Obstetrics Societies, Argentine Association of Clinical Oncology, Argentine Association of Clinical Oncology.
Civil society organizations			Non-governmental organizations that work for collective purposes, are autonomous and act on a not-for-profit basis.	Argentine League for the Fight Against Cancer (LALCEC)

MH: Ministry of Health, OS: “Obra social”

Any diffusion piece composed of text and/or images, created with the purpose of disseminating the breast cancer theme and including the institution’s logotype, was considered eligible.

The initial search was conducted by two investigators (SVG and MRT). Six institutions were randomly selected and independently assessed whether the total number of articles published by these institutions (n=206) met the inclusion criteria. For the 206 pieces identified, perfect concordance (Kappa=1, p < 0.001) between investigators was documented for compliance with the eligibility criteria. Given the optimal concordance, the remaining pieces were reviewed by a single investigator (SVG). Each piece was assigned an identifying number, and its degree of impact was documented (number of clicks on “like” and number of times it was shared).

### Procedures

*For the quantitative analysis*

Initially, we analyzed the pieces issued by 15 institutions (pilot test), and then all the pieces from the selected institutions. The analysis of each piece was carried out independently by a pair of researchers (SVG-MRT or SVG-MRY) through an online form. Out of a total of 2491 responses by each pair of researchers there was some degree of disagreement in 141 responses (5.6%), all resolved by consensus during a final meeting in which all of them participated. 

*For the qualitative analysis*

We selected the six pieces from each subgroup that had obtained the highest degree of repercussion (selection by quotas) and then analyzed the empirical corpus content (identification and interpretative analysis); the methodological proposal of Acal Diaz ^18^ for the analysis of still images was used as a reference, adapted specifically for this scenario through a problematization work carried out jointly by the whole team.

Based on the adapted categories, a pre-analysis of the empirical corpus was carried out with the intention of detecting emerging ideas and concepts, then a coding guide was created in an online form that allowed the uploading of examples and quotations. Based on the review of five pieces, two researchers (MAW and SVG) completed it independently (pilot test), trying to detect loading problems. Once the loading process was optimized, they continued with the remaining dissemination pieces. The information obtained independently by each of the two researchers was shared with a third researcher (SAT) in weekly meetings, during which (through an iterative process) concepts and ideas were identified from the analysis of the pieces, jointly constructing the emerging categories and subcategories, and preparing the final narrative report. The final sample size was subject to the criterion of theoretical saturation, and was reached with the 30 pieces initially selected.

### Variables

*For the quantitative analysis*

We analyzed whether each piece included recommendations on primary and secondary prevention of breast cancer, documenting the recommended screening methods, the starting and completion age and the proposed periodicity, and also evaluated the compliance of these recommendations with those of the INC. In addition, we analyzed whether the benefits and risks of screening were mentioned.

*For the qualitative analysis*

Five main aspects were analyzed: the structure and content of the diffusion pieces, the function of the image within the visual communication, the design of the campaign, the connotative resources used, and the relationship established between the piece and the receiver (supplementary material).

### Data analysis

*Quantitative analysis*

The analysis of the comlpiance of the data obtained with the INC recommendations was expressed as proportions.

### Ethical aspects

This study was approved (approval 4039) by the Ethics Committee for Research Protocols of the Hospital Italiano de Buenos Aires (IRB00010193).

## RESULTS

**Figure 1:**
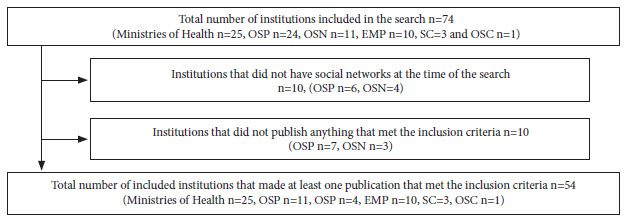
Flowchart outlining the process of searching for and identifying dissemination pieces linked to breast cancer published during October 2019 on the official sites of the 74 health institutions in Argentina included in our review.

Of the 74 institutions whose social media profiles were reviewed, 54 (73%) posted at least one breast cancer outreach piece during October 2019 (Figure 1), with a total of 171 pieces meeting the inclusion criteria.

### Quantitative aspect

We found that 46 of the 54 institutions (85%) made a recommendation on secondary prevention. Of these 46, 32 (69.5%) included mammography, alone or accompanied by other strategies, such as physical breast examination by a professional, which was also recommended by five institutions (10.8%) (Table 2).

**Table 2:** Strategies suggested by the 46 institutions that made recommendations on secondary prevention during the review of breast cancer outreach campaigns during October 2019 in Argentina.

Type of institution	n	Recommended by the INC		Not recommended by the INC		
		Mammography (%)	Physical examination of the breasts (%)	Breast self-examination (%)	Ultrasound (%)	Other ^a^ (%)
Health system in Argentina						
Ministries of health	22	18 (8.8)	4 (18.1)	7 (31.8)	1 (5.5)	11 (50.0)
Compulsory social insurance sector	13	9 (69.2)	1 (7.7)	4 (30.7)	1 (7.7)	8 (61.5)
Prepaid medicine companies	8	2 (25.0)	0 (0.0)	6 (75.0)	1 (12.5)	6 (75.0)
Scientific societies	2	2 (100.0)	0 (0.0)	1 (50.0)	1 (50.0)	2 (100.0)
Civil society organizations	1	1 (100.0)	0 (0.0)	0 (0.0)	0 (0.0)	1 (100.0)
Total	46	32 (69.5)	5 (10.8)	18 (39.1)	4 (8.7)	28 (60.8)

The same institution may have recommended more than one strategy for secondary prevention of breast cancer, so the sum of the percentages may exceed 100%.

a Others: consulting a general practitioner (n=12), gynecologist (n=4), mastologist (n=2), and unspecified “medical check-ups” (n=13).

Among the recommendations that did not comply with the INC, breast self-examination was mentioned by 18 institutions (39%), five of them on a monthly basis. In eight institutions (17%) it was the only screening strategy recommended, while breast ultrasound was recommended by four institutions (8.7%).

As shown in Table 3, of the 32 institutions that recommended mammography, a total of 22 (69%) specified the starting age and only 9 (28%) the age of screening completion, and 20 (63%) of them specified the recommended periodicity. 

The National Ministry of Health was the only institution that issued a recommendation in complete agreement with that of the INC.

**Table 3:** Recommendations on the ages of beginning, completion, and periodicity of screening with mammography reported by 32 institutions published during October 2019 in the official social networks of institutions in Argentina.

Type of institution	n	Recommendation by age							
		Beginning (years)			Completion (years)		Periodicity		
		NC	35-45	50 ^a^	NC	65-70 ^a^	NC	Annual	Biennial ^a^
Health system in Argentina									
Ministries of health	18	5 (27.8)	6 (33.3)	7 (38.9)	12 (66.7)	6 (33.3)	8 (44.4)	7 (38.9)	3 (16.7)
Compulsory social insurance sector	9	4 (44.5)	3 (33.3)	2 (22.2)	7 (77.8)	2 (22.2)	3 (33.3)	4 (44.5)	2 (22.2)
Prepaid medicine companies	2	1 (50.0)	1 (50.0)	0 (0.0)	2 (100.0)	0 (0.0)	1 (50.0)	1 (50.0)	0 (0.0)
Scientific societies	2	0 (0.0)	2 (100.0)	0 (0.0)	2 (100.0)	0 (0.0)	0 (0.0)	2 (100.0)	0 (0.0)
Civil society organizations	1	0 (0.0)	1 (100.0)	0 (0.0)	0 (0.0)	1 (100.0)	0 (0.0)	1 (100.0)	0 (0.0)
Total	32	10 (31.2)	13 (40.7)	9 (28.1)	23 (71.9)	9 (28.1)	12 (37.5)	15 (46.9)	5 (15.6)

NC: not clarified.

a Recommendations comply with the National Cancer Institute.

Of the 54 analyzed institutions, 44 (81%) mentioned benefits of screening and only three expressed them quantitatively (two of them in relative terms and one in absolute terms). Among the clinically relevant benefits, the increased chance of cure was the most mentioned one (42%) followed by decreased mortality (18%), increased survival (11%) and early or less invasive treatment (14.8%). In contrast, 6 institutions (11%) mentioned “prevention” as a benefit of screening and 19 (35%) mentioned early diagnosis as a benefit in itself (Table 4). No institution mentioned possible harms of screening.

**Table 4:** Benefits argued to support the convenience of performing breast cancer screening through the strategies proposed by 54 institutions in Argentina during October 2019.

Type of institution	n	Increase in probability of cure (%)	Mortality reduction (%)	Increase in survival (%)	Early / less invasive treatment (%)	**Early detection as a benefit *per se* (%)**	Prevention as a goal in itself (%)
Health System in Argentina							
Ministries of health	25	10 (40.0)	4 (16.0)	0 (0.0)	3 (12.0)	9 (36.0)	3 (12.0)
Compulsory social insurance sector	15	5 (33.3)	2 (13.3)	2 (13.2)	2 (13.3)	5 (33.3)	3 (20.0)
Prepaid Medicine Companies	10	5 (50.0)	2 (20.0)	2 (20.0)	2 (20.0)	2 (20.0)	0 (0.0)
Scientific societies	3	3 (100.0)	1 (33.3)	2 (66.6)	1 (33.3)	2 (66.6)	0 (0.0)
Civil Society Organizations	1	0 (0.0)	1 (100.0)	0 (0.0)	0 (0.0)	1 (100.0)	0 (0.0)
Total	54	23 (42.6)	10 (18.5)	6 (11.1)	8 (14.8)	19 (35.1)	6 (11.1)

A single institution may have argued more than one benefit of breast cancer screening, so the sum of percentages may exceed 100%.

The pieces from 6 of the 54 institutions (11%) mentioned an endorsement or entity cited as a reference and/or source of information for the recommendation issued, which in all cases were not consistent with those of the INC (data not shown).

### Qualitative aspect

Thirty dissemination pieces (supplementary material) were analyzed and the main emerging categories (and their subcategories) were identified, which are described below. 

*a. Images and design of the pieces*

The images were classified according to their function within the piece and the content of the message they convey.

The exhibition of naked female bodies was a widely used strategy to attract attention, pieces 1 and 2 of the supplementary material (SM). On the other hand, those that seek to create identification with the receiver do so by showing women with stereotyped bodies according to the hegemonic “ideal” of current beauty (pieces 1 and 3 of the SM), or also by representing the “fighting” woman, using images of scarves, a symbol of empowerment and the vindication of women’s rights (piece 5 of the SM). In addition, the images support the information provided by the text (pieces 4 and 6 of the SM), and sometimes only have an aesthetic purpose (pieces 8 and 9 of the SM) showing bodies reminiscent of works of art (pieces 10, 11 and 12 of the SM).

*Content of the message conveyed by the image (gender and beauty stereotypes)*

There is a predominance of images of slim and young women, outside the recommended screening age (pieces 1, 14 and 16 of the SM), and with characteristics that reflect the currently most socially accepted beauty stereotype (piece 13 of the SM). There is only one piece that shows diversity in the women represented (SM piece 4). The women appear to be healthy, without showing women undergoing oncological treatments or in advanced stages of the disease (piece 13 of the SM). Many pieces show women without faces, focusing the attention on their hands, torso (often naked), or only on one breast (pieces 12, 14 and 15 of the SM), and often showing poses that give the image an erotic connotation (piece 1 of the SM).

Concepts that reinforce the stereotype of the feminine gender prevail, the color pink, cursive letters, and the use of hearts or flowers predominate (pieces 17, 18 and 30), and the woman is shown with a passive, smiling and delicate attitude (pieces 16, 19 and 20). 

*b. The text*

The textual elements that make up the diffusion pieces fulfill various functions: awareness-raising, persuasion and transmission of information.

*Awareness and persuasion*

Textual elements aim to consolidate the concept of October as a month dedicated to breast cancer (pieces 19, 20 and 21 of the SM). We observed that persuasion strategies are usually applied by asking the recipient “When was your last check-up?” and affirming “Prevention is in your hands” (pieces 5, 11 and 22 of the SM). Some pieces include warning signs or false beliefs about breast cancer (SM pieces 4 and 23).

*Transmitted (Dis) information*

The lack of message segmentation by age, the promotion of practices without scientific support, the overestimation of their benefits and the omission of risks derived from their implementation are prevalent. The messages are usually addressed to women of all ages; the question “Have you already had your annual studies?” seems to convey that screening should be performed at any age without distinction (piece 21 of the SM).

A large number of the pieces highlight the importance of prevention with high-impact phrases such as “Prevention saves lives” (pieces 10, 24 and 25 of the SM), feeding the false idea that the development of all cancers could be avoided by performing timely studies. On the other hand, many campaigns recommend breast self-examination, without evidence to support it, since it has not been found that its implementation reduces mortality ^19^. Even so, slogans such as “Today your hand saves lives” and “Touch them once a month” provide counseling without scientific support (parts 3 and 8 of the SM).

There was a tendency to overestimate the benefits of screening with statements such as “A study in time can save your life” (item 1 of the SM) without mentioning the extent to which screening reduces mortality. On the other hand, in statements such as “Don't give up, you can do it” (SM item 25), the idea that cancer can always be cured or that its evolution is conditioned by the behaviors adopted prevails.

*c. The images, the design of the piece and the text: feelings and emotions evoked by the diffusion pieces.*

Both the images and the information conveyed by the textual elements can evoke various feelings and emotions in the receiving audience. 

Many campaigns evoke commitment and unity. Phrases such as “For me, for you, for all of us”, “Today we unite against breast cancer”, convey that the “fight” is collective, as a phenomenon of social responsibility (pieces 4, 7 and 8 of the SM).

On the other hand, there is a tendency to make women responsible by affirming that “Prevention is in your hands”, (part 1 of the SM) which, by assuring that cancer can be prevented and that doing so depends on the woman herself, can generate guilt and stigmatization in those who have not carried out the recommended studies or who have received a diagnosis of cancer.

Some pieces evoke concern and fear by highlighting “1 in 8 women can suffer breast cancer, the older the woman, the higher the risk” (SM piece 27), while other pieces convey hope “A study in time can save your life” (SM piece 27). It is common for both messages to coexist in the same campaign, first stating that the risk is “high” and then recommending studies to “avoid it” (piece 7 of the SM).

The importance of empowerment prevails, defined by these campaigns as a way of taking responsibility for one’s own health, and of carrying out the recommended studies (item 5).

On the other hand, heroism is referred to in women who manage to be cured by using epic language. There is talk of “Fighting”, “Beating this disease” (pieces 7, 24 and 26 of the SM), which implies that the results depend on individual strength and will and does not consider other variables, such as the characteristics of the type of cancer ^20^ or the social determinants of the patient.

Many pieces also convey a notion of obligatory nature: “Have you already undergone the annual studies?”, “Don't give up”, assuming that there is only one valid option: to undergo studies and then treatment. We also observed that recommendations are usually formulated in an imperative way: “Have your first mammogram at 35 years of age” (pieces 3, 16 and 25 of the SM).

## DISCUSSION

After quantitatively analyzing the breast cancer campaigns published in Argentina in October 2019, we found a predominance of messages that do not comply with the INC, like the advice to perform mammography at earlier ages and/or with more frequent intervals, and/or to omit the age of completion or to perform breast self-examination. Although it is desirable for women to detect warning signs, self-examination should not be considered a screening strategy because it can lead to false reassurance ^19^.

At the same time, the campaigns only communicated the potential benefits of screening without mentioning potential harms. In the few pieces that quantified the benefits, they did so by using measures of effect in relative terms, when it is advisable to state them in absolute terms as they convey a clearer idea of the potential benefit of having a mammogram ^21^.

This bias in campaigns in favor of screening disregards the growing evidence that questions its real effectiveness and warns of its potential harms ^10^. It also generates false expectations since it assumes that the disease has a linear and predictable behavior, both preventable and curable through the recommended strategies, which is a falsely simplified interpretation of its biological behavior, and hides the wide spectrum of its potential evolution ^20^ ^,^ ^22^.

Finally, it is striking that a national and official entity such as the INC, which has solidly documented recommendations on breast cancer, is not cited as a source. From the qualitative point of view, the reinforcement of gender and beauty stereotypes and the imposition of a hegemonic path as the only valid way to go through the health-disease process stand out.

The prevailing concept of femininity is based on a binary and heteronormative gender system in which women are linked to stereotypes such as the color pink, delicate and passive ^23^ ^,^ ^24^, which could act as barriers to the reception of information and access to health care for groups that do not feel represented by these messages.

Likewise, images of naked bodies and in provocative poses perpetuate the sexualization and objectification of women ^25^ and reinforce certain canons of beauty in which thinness and youth are imposed as values ^26^, findings that coincide with research carried out in other countries ^26^ ^,^ ^27^.

Thirdly, in the analyzed campaigns, the imposition of a single valid option for the health-disease process prevails, through the performance of screening tests and eventual treatments.

The analyzed messages reduce this process to individual responsibility, exclude economic, political and cultural factors ^28^, and blame and stigmatize those women who decide not to undergo screening, who are unable to access it, or even those who are diagnosed with cancer ^20^. A message of “feminine empowerment” is sent out, but from a normative point of view that appeals to fear and guilt as tools of persuasion, and based on the hegemonic medical model ^29^ ^,^ ^30^.

Likewise, the image of the woman who decides to “fight against cancer” is exalted through metaphors of war and the comparison of the disease with a battle that turns the woman who has been cured into a “winner”, which implies that it depends on the woman’s attitude and coping style ^30^. This creates the image of a strong, empowered, fighting woman (she-ro: she is a hero) ^24^. The flip side is represented by women who are going through the disease, or who die from it, who are made invisible or who, even with the use of these metaphors, are seen as having lost or “failed”, a situation that has also been documented in Spain ^20^.

One of the limitations of this study is that the universe defined as a cut-off for this research was limited to the analysis of the diffusion pieces issued by institutions linked to health care, without evaluating the messages issued by other types of opinion leaders such as celebrities (for example, from the artistic or sports environment), whose opinions and life stories can exert very powerful influences on those who receive them.

Although there is literature on this subject ^13^ ^,^ ^31^, no other studies have been found that have analyzed the information transmitted by diffusion campaigns on breast cancer in Argentina. We consider that this research is the first step to propose a key point to focus the efforts on providing accurate communication to women, with clear information restricted to recommendations with high quality scientific support.

Inaccurate and incomplete information hinders women from making a correct assessment of their risks and benefits, and from making a free and informed decision about whether or not to undergo a screening test. We consider it essential to move towards a model of shared decision-making in which women are involved in the decision whether or not to undergo a medical intervention and their values and preferences are considered ^32^. This approach empowers the patient, who takes an active and responsible role in her health decisions, with realistic expectations and, therefore, improves the probabilities of achieving the most desirable results ^33^.

In conclusion, the messages issued about breast cancer screening in October 2019 by the included institutions were mostly inconsistent with the INC. This occurred at the expense of messages recommending mammograms at younger ages, at more frequent intervals, and the implementation of strategies that did not prove to decrease mortality, such as breast self-examination. Likewise, there was a tendency to overestimate the benefits of screening and every case failed to mention potential harms associated with its implementation.

On the other hand, the analyzed messages present breast cancer as a linear and homogeneous disease whose natural course could be strongly modified by individual actions, which is a falsely simplified interpretation, and generates confusion and false expectations.

Finally, we found that these campaigns attempt to address one problem, but accentuate others, such as the perpetuation of gender and beauty stereotypes, the hegemonic medical model, and the blaming and stigmatization of those who do not follow the imposed norm.

We consider it appropriate to rethink the focus of campaigns related to breast cancer. We propose that they should be spaces where complete and balanced information is conveyed, which should make the possible benefits and risks of screening or not screening transparent according to the best available evidence, as well as favoring diversity and putting an end to the perpetuation of gender and beauty stereotypes.
