# Supplementary material for: Quantitative and qualitative analysis of Argentine breast cancer prevention campaigns disseminated by still images on social networks during October 2019
Source: Rev Peru Med Exp Salud Publica. 2022 Jun 30;39(2):152–60. doi: 10.17843/rpmesp.2022.392.11019 (PMC11397677; doi:10.17843/rpmesp.2022.392.11019)

Anexo de piezas de difusión

Anexo 3.3

Pieza 21

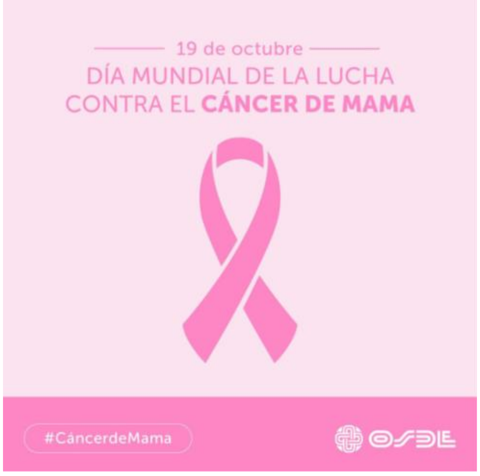

Pieza 22

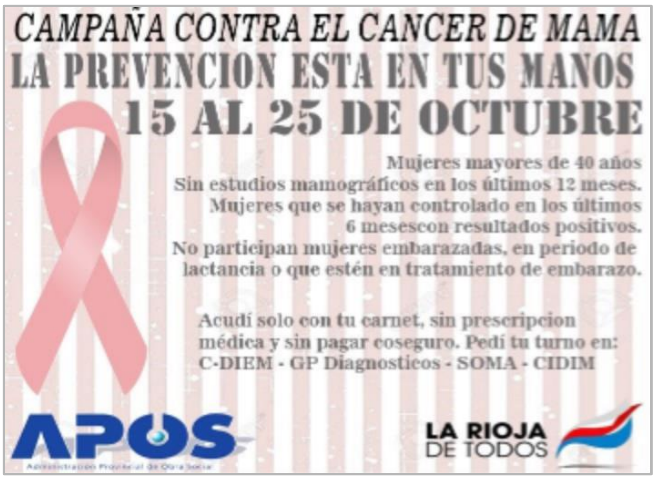

Pieza 23

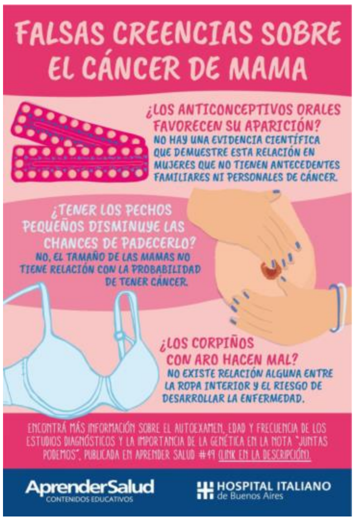

Pieza 24

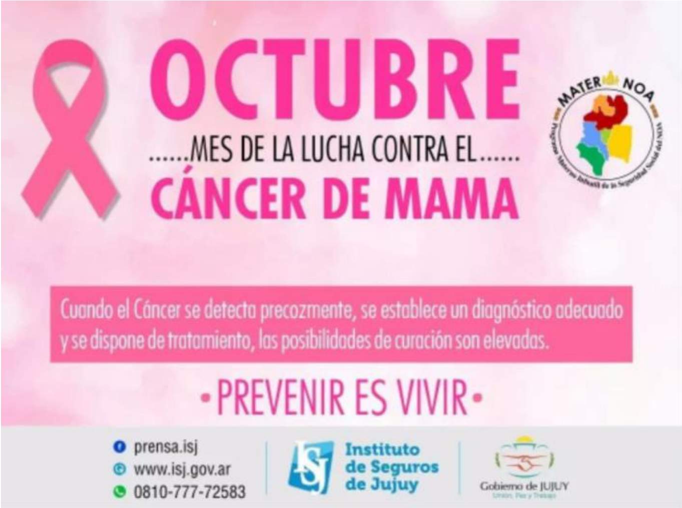

Pieza 25

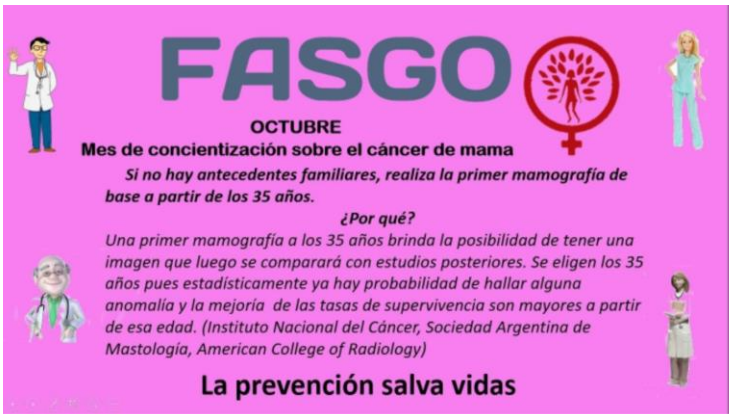

Pieza 26

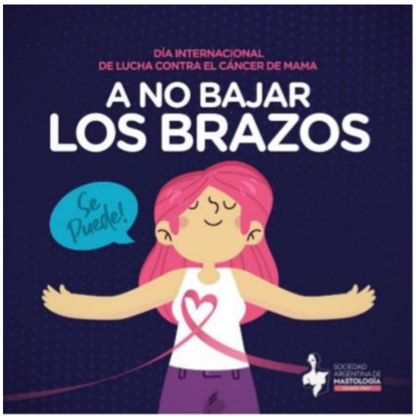

Pieza 27

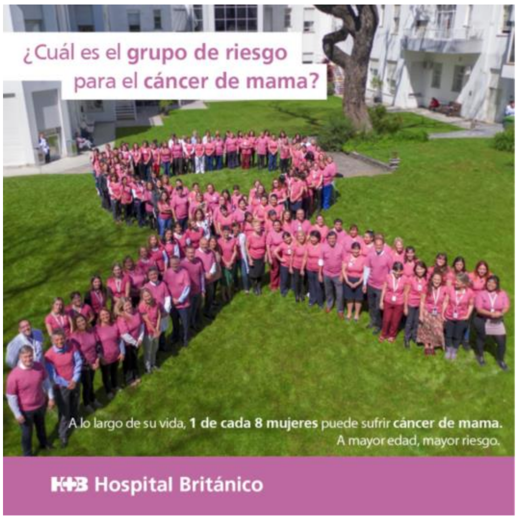

Pieza 28

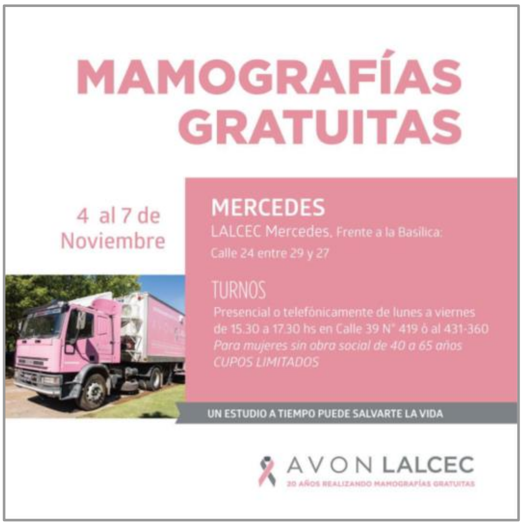

Pieza 29

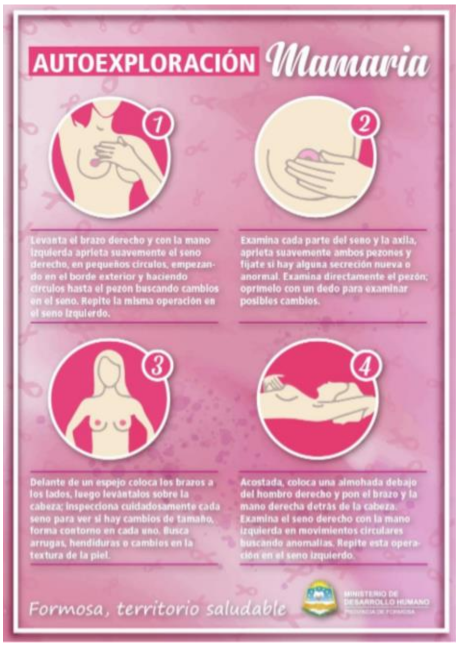

Pieza 30

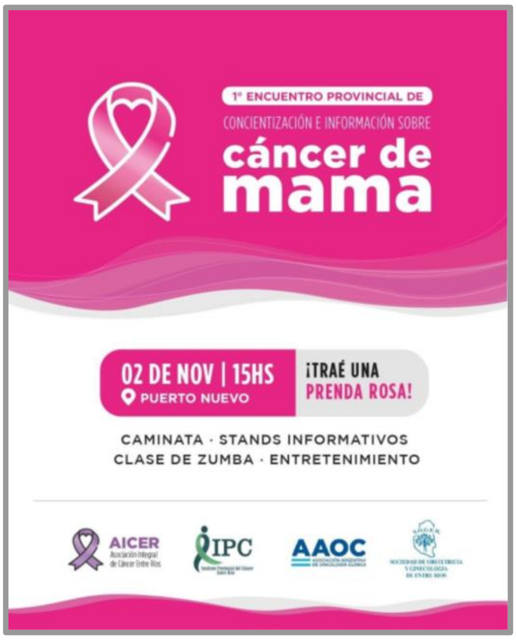

Supplement: Supplementary material. — Available in the electronic version of the RPMESP. [file rpmesp-39-02-11019-s001.zip › Anexo 3.3.pdf]
