# Supplementary figures and images for: Quantitative and qualitative analysis of Argentine breast cancer prevention campaigns disseminated by still images on social networks during October 2019
Source: Rev Peru Med Exp Salud Publica. 2022 Jun 30;39(2):152–60. doi: 10.17843/rpmesp.2022.392.11019 (PMC11397677; doi:10.17843/rpmesp.2022.392.11019)

# **Anexo de piezas de difusión**

## **Anexo 3.1**

Pieza 1

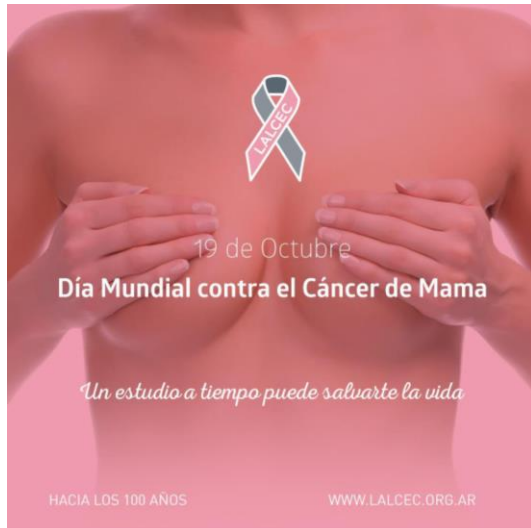

Pieza 2

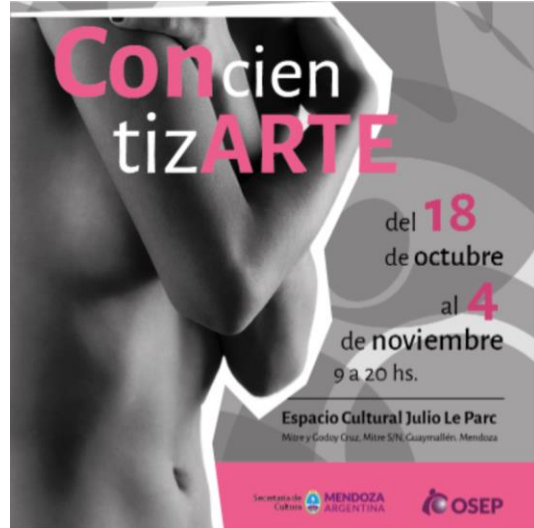

Pieza 3

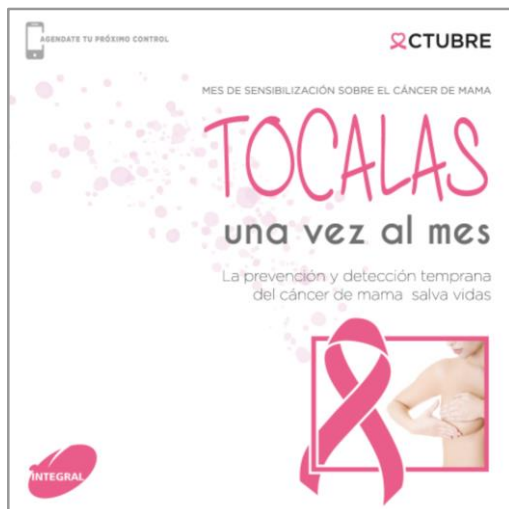

Pieza 4

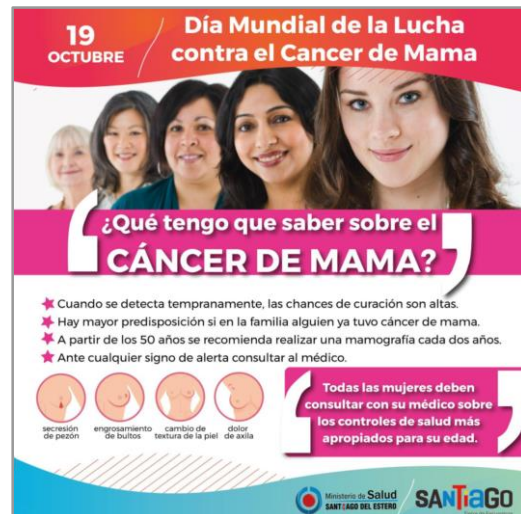

Pieza 5

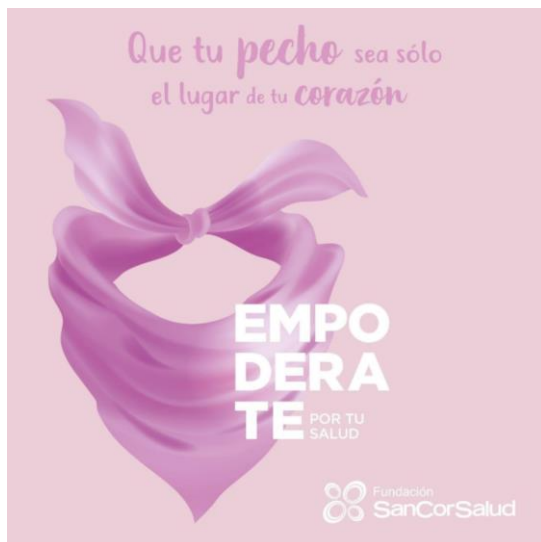

Pieza 6

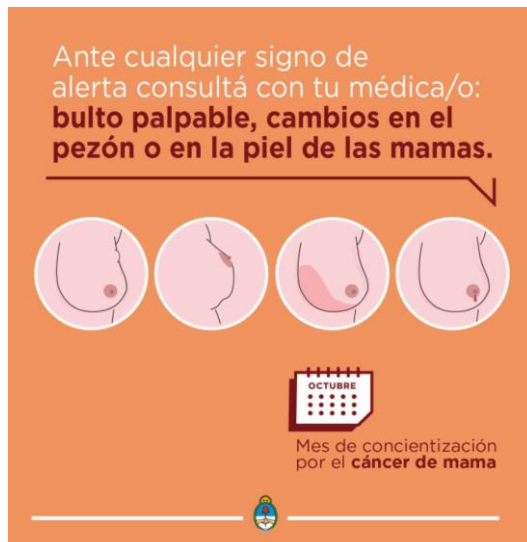

Pieza 7

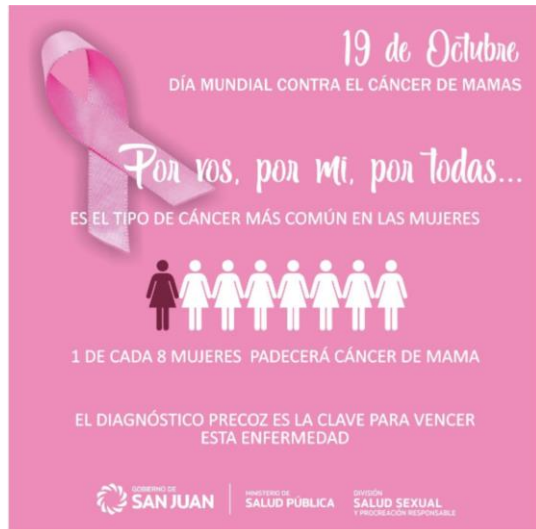

Pieza 8

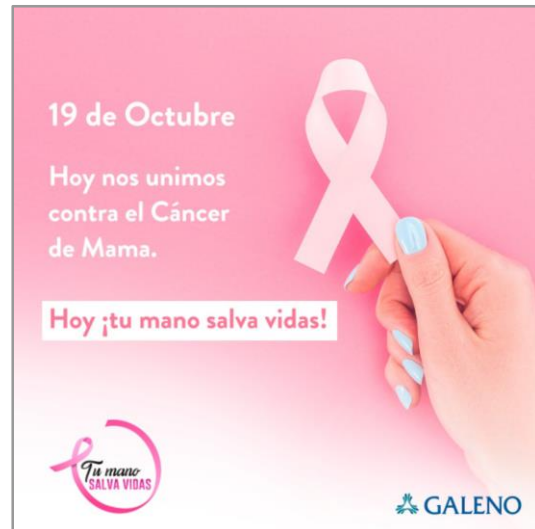

Pieza 9

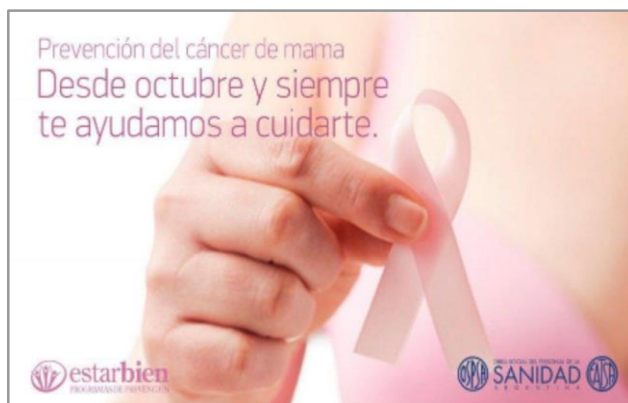

Pieza 10

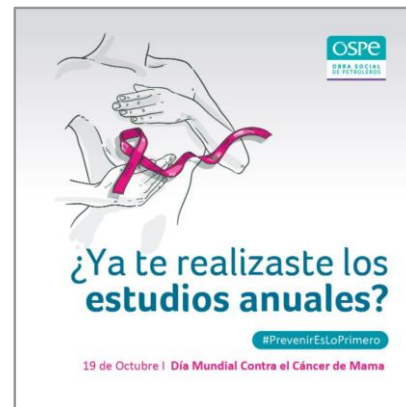

Supplement: Supplementary material. — Available in the electronic version of the RPMESP. [file rpmesp-39-02-11019-s001.zip › Anexo 3.1.pdf]

# **Anexo de piezas de difusión**

## **Anexo 3.2**

Pieza 11

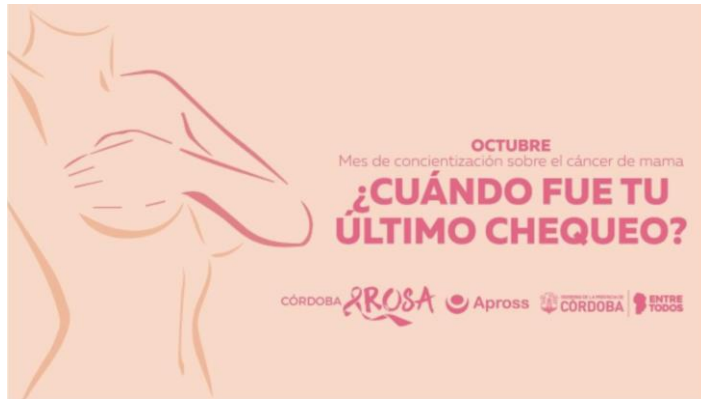

Pieza 12

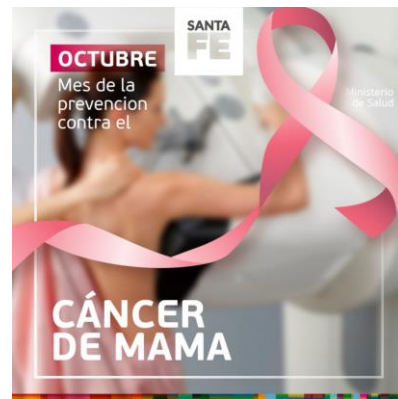

Pieza 13

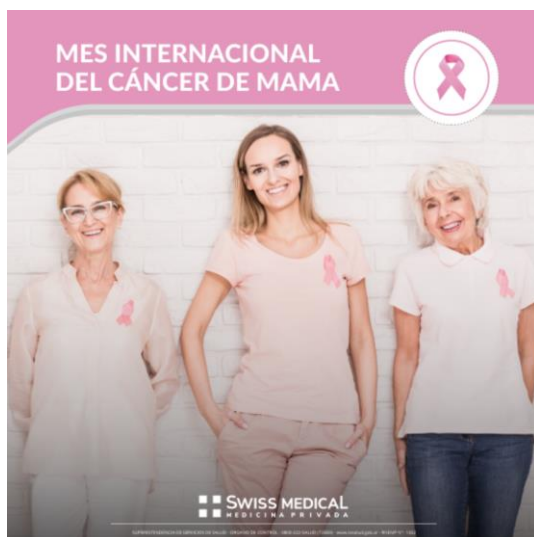

Pieza 14

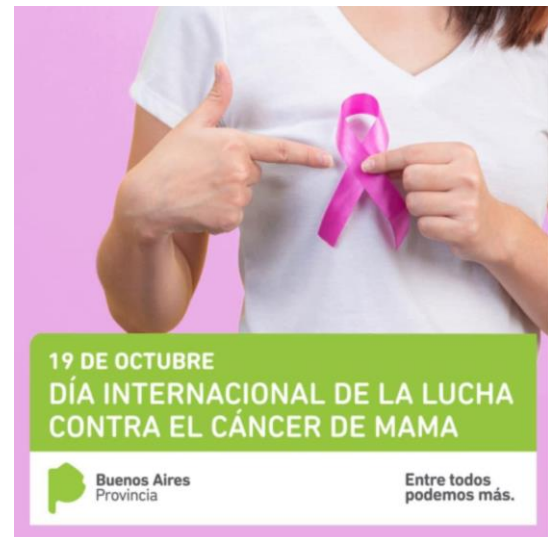

Pieza 15

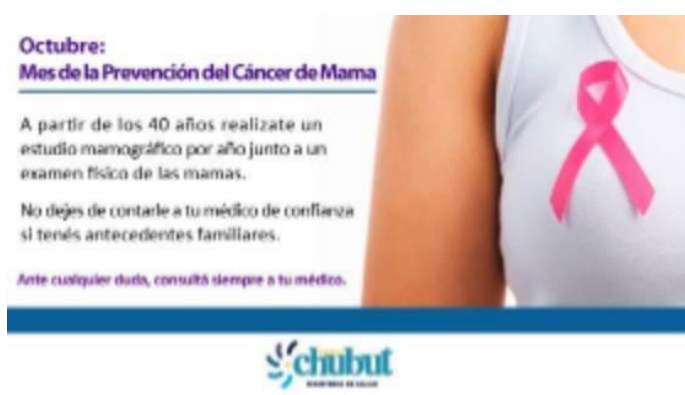

Pieza 16

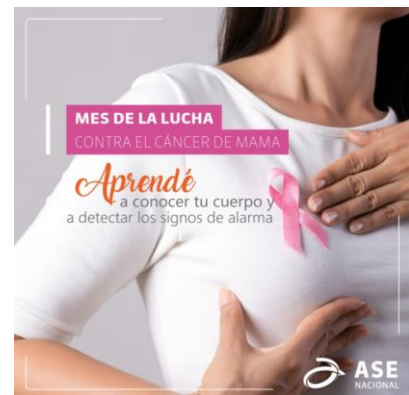

Pieza 17

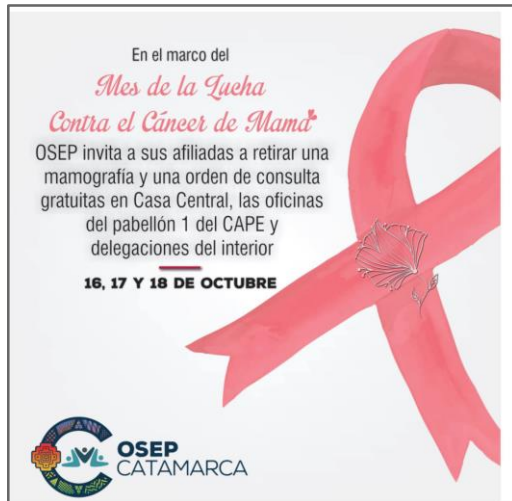

Pieza 18

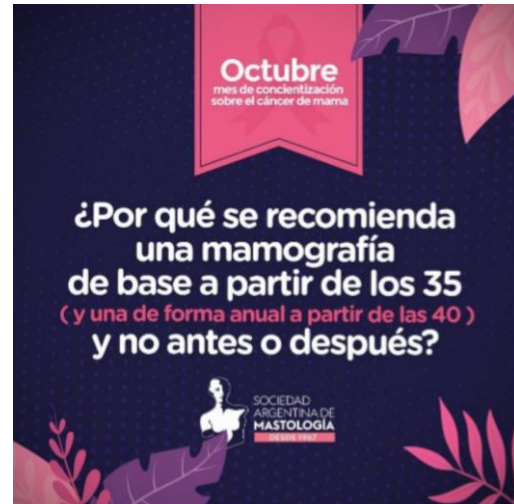

Pieza 19

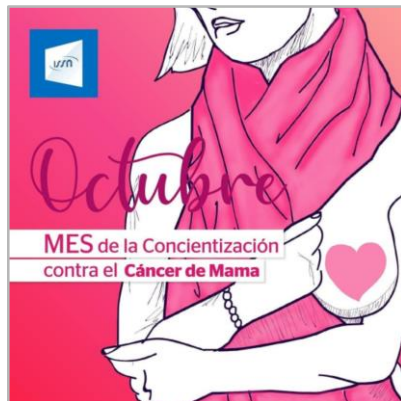

Pieza 20

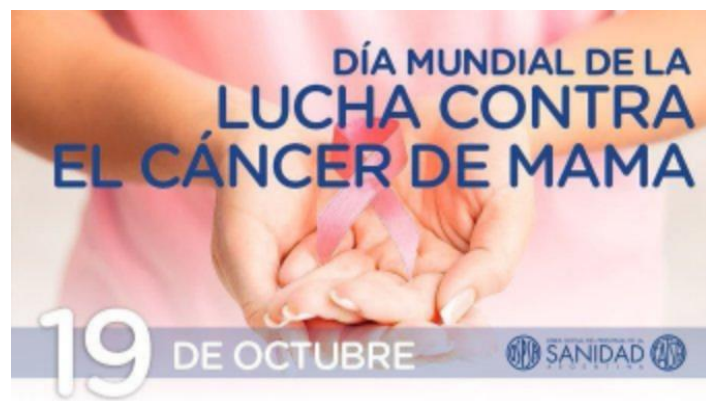

Supplement: Supplementary material. — Available in the electronic version of the RPMESP. [file rpmesp-39-02-11019-s001.zip › Anexo 3.2.pdf]
