# Supplementary material for: Quantitative and qualitative analysis of Argentine breast cancer prevention campaigns disseminated by still images on social networks during October 2019
Source: Rev Peru Med Exp Salud Publica. 2022 Jun 30;39(2):152–60. doi: 10.17843/rpmesp.2022.392.11019 (PMC11397677; doi:10.17843/rpmesp.2022.392.11019)
Supplement: Translation

# Translation: Análisis cuantitativo y cualitativo de las campañas argentinas de prevención de cáncer de mama difundidas mediante imágenes fijas en redes sociales durante octubre del 2019

**Authors:** Sofía Victoria Gürtler, Manuel Rodríguez-Tablado, Mariela Alejandra Weisbrot, María Victoria Ruiz-Yanzi, Karin Silvana Kopitowski, Sergio Adrián Terrasa

**Affiliations:** 1 Hospital Italiano de Buenos Aires, Ciudad Autónoma de Buenos Aires, Argentina.; 2 Universidad Nacional de Lanús, Remedios de Escalada, Buenos Aires, Argentina.

**Keywords:** Neoplasias de la Mama, Mamografía, Tamizaje Masivo, Medios de Comunicación, Concientización, Difusión de Información, Redes Sociales

## Abstract

**Objetivos.:**

Documentar cuantitativamente el grado de concordancia con las recomendaciones del Instituto Nacional del Cáncer (INC) de los mensajes institucionales emitidos en redes sociales de Argentina durante octubre de 2019, en el contexto de las campañas de prevención del cáncer mamario, y analizar cualitativamente los elementos icónicos y textuales que conforman sus piezas de difusión.

**Materiales y métodos.:**

Análisis cuantitativo y cualitativo de 171 piezas de difusión emitidas durante octubre de 2019 por 54 instituciones, a partir de la evaluación de su concordancia con las recomendaciones del INC, la descripción de las principales recomendaciones discordantes (análisis cuantitativo) y el análisis cualitativo de 30 piezas.

**Resultados.:**

Ninguno de los mensajes emitidos mencionó potenciales daños del tamizaje. Solamente los del Ministerio de Salud de la Nación fueron totalmente concordantes con las recomendaciones del INC, mientras que los restantes recomendaban realizar mamografías a edades más tempranas o a intervalos más breves. El autoexamen mamario fue la recomendación más frecuente entre las discordantes. Predominaron las imágenes de cuerpos femeninos vinculadas con los estereotipos predominantes de género y belleza, y los discursos paternalistas que apelan al miedo y a la culpa.

**Conclusiones.:**

Los mensajes emitidos en las piezas de difusión analizadas no fueron concordantes con las recomendaciones del INC, a pesar de que estas últimas están respaldadas por evidencia científica. Por otro lado, los mensajes refuerzan los estereotipos de género y belleza, la culpa y el modelo médico-hegemónico.

## INTRODUCCIÓN

El Instituto Nacional del Cáncer (INC) de Argentina recomienda a las mujeres de 50 a 70 años realizar, al menos, una mamografía cada dos años, asociada a un examen mamario realizado por un profesional de la salud ^1^, y personalizar la decisión en las mujeres de 40 a 50 años y en las mayores de 70 ^2^. Estas recomendaciones son concordantes con las de la Organización Mundial de la Salud y los Servicios Preventivos Norteamericano y Canadiense ^3^ ^-^ ^5^.

El tamizaje de cáncer mamario acarrea potenciales daños (falsos positivos, sobrediagnóstico y sobretratamiento) y sus beneficios no son tan claros como inicialmente se pensaba ^6^ ^-^ ^8^. Se estima que, si 2000 mujeres se realizaran mamografías durante diez años, se evitaría la muerte de una, y en diez de ellas ocurriría sobrediagnóstico y/o sobretratamiento ^9^. Asimismo, en 1980 la probabilidad de que una mujer recibiera un diagnóstico de cáncer mamario a lo largo de su vida era de una en cada doce y en la actualidad esa cifra ha ascendido a una en cada ocho ^10^. Además, desde el inicio del tamizaje con mamografía en EE. UU., aumentó el diagnóstico de cáncer mamario sin una disminución semejante en la mortalidad por esta causa ^11^, lo que podría explicarse por un aumento de la detección en estadios tempranos sin un descenso semejante en la incidencia de enfermedad avanzada y/o metastásica. Sin embargo, investigaciones internacionales ^12^ y en Argentina ^13^ han documentado que los mensajes predominantes en los medios de comunicación tienden a sobreestimar los beneficios del tamizaje y a invisibilizar sus potenciales riesgos.

En Argentina, en octubre de cada año, varias instituciones de salud, sociedades científicas y organizaciones de la sociedad civil emiten mensajes a gran escala sobre el cáncer mamario. Está documentado que la información que circula masivamente en medios de comunicación puede influir en la formación de opinión y en la toma de decisiones por parte de la población ^14^ ^,^ ^15^.

Sin embargo, no se contaba hasta ahora con una evaluación sistemática del contenido de estas campañas en Argentina.

Este trabajo tuvo dos objetivos: 1) documentar cuantitativamente el grado de concordancia de los mensajes institucionales emitidos en las redes sociales de Argentina durante octubre de 2019 en el contexto de las campañas de prevención del cáncer mamario con las recomendaciones del INC, y 2) analizar cualitativamente los elementos icónicos y textuales de sus piezas de difusión.

**MENSAJE CLAVE**

Motivación para realizar el estudio: la temática del cáncer mamario es ampliamente difundida en campañas masivas. Como tamizaje, el Instituto Nacional del Cáncer (INC) recomienda realizar una mamografía cada dos años entre los 50 y 70 años. Esta información no parece ser transmitida a la población general.

Principales hallazgos: solamente los mensajes emitidos por el Ministerio de Salud de la Nación fueron concordantes con el INC. La promoción del autoexamen mamario fue el más frecuente entre los discordantes, y a su vez se vieron reforzados estereotipos de género y belleza y el modelo médico-hegemónico.

Implicancias: Es necesario repensar el diseño de las campañas para evitar la difusión de información errónea, y los discursos paternalistas donde, además, se perpetúan estereotipos de género y belleza.

## MATERIALES Y MÉTODOS

### Diseño del estudio

Estudio de corte transversal a través de técnicas de análisis cuantitativo y cualitativo.

### Criterios de selección

Se seleccionaron las tres redes sociales abiertas con contenidos de imagen fija con mayor número de usuarios regulares en Argentina ^16^: Facebook, Instagram y Twitter. 

Dada la naturaleza fragmentada del sistema de salud de Argentina, fueron identificadas las piezas de difusión publicadas por representantes de los sectores estatal, privado y de la seguridad social en Facebook, Instagram y Twitter, entre el 1 al 31 de octubre del 2019. Fueron incluidas las piezas emitidas por el Ministerio de Salud de la Nación y los ministerios de las 24 provincias, las 24 obras sociales provinciales, las once obras sociales nacionales y las diez empresas de medicina prepaga con mayor número de afiliados ^17^ (Tabla 1). También se incluyeron aquellas sociedades científicas y organizaciones de la sociedad civil vinculadas con la temática del cáncer mamario.

**Tabla 1:** Descripción de las instituciones cuyas piezas de difusión integraron la muestra de la investigación.

Tipo de institución			Descripción	Instituciones elegidas
Sistema de Salud en Argentina	Ministerios de Salud		Financiado con recursos provenientes de las rentas generales. Integrado por las estructuras administrativas provinciales y nacionales de nivel ministerial, y la red de hospitales y centros de salud públicos que prestan atención gratuita a toda la población	MS de la Nación, MS de Ciudad Autónoma de Buenos Aires (CABA), MS de Buenos Aires, MS de Catamarca, MS de Chaco, MS de Chubut, MS de Córdoba, MS de Corrientes, MS de Entre Ríos, MS de Formosa, MS de Jujuy, MS de La Pampa, MS de La Rioja, MS de Mendoza, MS de Misiones, MS de Neuquén, MS de Rio Negro, MS de Salta, MS de San Luis, MS de San Juan, MS de Santa Cruz, MS de Santa Fe, MS de Santiago del Estero, MS de Tierra del Fuego, MS de Tucumán
	Sector del Seguro Social Obligatorio	Provinciales	Financiado mediante el aporte y la contribución obligatorios de trabajadores y empleadores e integrado por las «Obras Sociales» (OS). Brinda cobertura a los trabajadores asalariados y sus familias, según ramas de actividad (OS Nacionales). A su vez, cada provincia cuenta con una OS que brinda cobertura a los empleados públicos de su jurisdicción (OS Provinciales).	OS de CABA, OS de Buenos Aires, OS de Catamarca, OS de Chaco, OS de Chubut, OS de Córdoba, OS de Corrientes, OS de Entre Ríos, OS de Formosa, OS de Jujuy, OS de La Pampa, OS de La Rioja, OS de Mendoza, OS de Misiones, OS de Neuquén, OS de Rio Negro, OS de Salta, OS de San Luis, OS de San Juan, OS de Santa Cruz, OS de Santa Fe, OS de Santiago del Estero, OS de Tierra del Fuego, OS de Tucumán
		Nacionales		Programa de Asistencia Médica Integral (PAMI), Empleados de comercio y actividades civiles (OSECAC), Personal de la construcción (OSPECON), Unión del personal civil de la nación (OSPCN), Personal del turismo hotelería y gastronomía (OSUTHGRA), Petroleros (OSPE), Unión obrera metalúrgica de la república argentina (OSUOMRA), Acción social de empresarios (ASE), Personal de la sanidad argentina (OSPSA), Obra social del personal del organismo de control externo (OSPOCE).
	Empresas de medicina prepaga		Financiado por dinero de individuos particulares y/o empresas. Incluye a las entidades de seguro voluntario llamadas Empresas de Medicina Prepaga cuyos servicios las personas y las familias pagan en forma de cuotas fijas mensuales.	Organización de Servicios Directos Empresarios (OSDE), Swiss Medical, Galeno, Omint, Medicus, Accord Salud, Medifé, Sancor Salud, Hospital Italiano, Hospital Británico.
Sociedades científicas			Asociaciones de profesionales, investigadores o especialistas reunidos en torno a un área del conocimiento, con el objetivo de facilitar descubrimientos científicos y diseminar este conocimiento.	Sociedad Argentina de Mastología, Federación Argentina de Sociedades de Ginecología y Obstetricia, Asociación Argentina de Oncología Clínica
Organizaciones de la sociedad civil			Organizaciones no gubernamentales que trabajan para fines colectivos, son autónomas y actúan sin fines de lucro	Liga Argentina de la Lucha Contra el Cáncer (LALCEC)

MS: Ministerio de Salud, OS: Obra social

Se consideró elegible toda pieza de difusión compuesta por texto y/o imágenes, creada con el fin de difundir la temática del cáncer mamario que incluyera el isologo de la institución.

La búsqueda inicial fue realizada por dos investigadores (SVG y MRT). Se eligieron al azar seis instituciones y se evaluó de forma independiente si el total de las piezas publicadas por esas instituciones (n=206) cumplía los criterios de inclusión. Se documentó para las 206 piezas identificadas una perfecta concordancia (Kappa=1, p < 0,001) entre los investigadores respecto del cumplimiento de los criterios de elegibilidad. Dada la óptima concordancia, el resto de las piezas fueron revisadas por una única investigadora (SVG). A cada pieza se le asignó un número identificatorio, y se documentó su grado de repercusión (número de clics en «me gusta» y número de veces que fue compartida).

### Procedimientos

*Para el análisis cuantitativo*

Inicialmente se analizaron las piezas emitidas por 15 instituciones (prueba piloto), y se prosiguió con la totalidad de las piezas de las instituciones seleccionadas. El análisis de cada pieza fue realizado en forma independiente por una pareja de investigadores (SVG-MRT o SVG-MRY) a través de un formulario en línea. De un total de 2491 respuestas por cada pareja de investigadores hubo algún grado de desacuerdo en 141 respuestas (5,6%), todos resueltos por consenso durante una reunión final en la que participaron todos ellos. 

*Para el análisis cualitativo*

Se seleccionaron las seis piezas de cada subgrupo que habían obtenido el mayor grado de repercusión (selección por cuotas), sobre las que se realizó un análisis de contenido del corpus empírico (análisis identificativo e interpretativo), y se utilizó como referencia la propuesta metodológica de Acal Diaz ^18^ para el análisis de imágenes fijas, adaptada exprofeso para este escenario a través de un trabajo de problematización realizado de manera conjunta por todo el equipo.

En base a las categorías adaptadas se realizó un preanálisis del corpus empírico con la intención de detectar ideas y conceptos emergentes, y se creó una guía de codificación en un formulario en línea que permitía cargar ejemplos y citas. A partir de la revisión de cinco piezas, dos investigadoras (MAW y SVG) lo completaron en forma independiente (prueba piloto), procurando detectar problemas de carga. Una vez optimizado el proceso de carga, continuaron con las restantes piezas de difusión. La información obtenida en forma independiente por cada una de las dos investigadoras fue compartida con un tercer investigador (SAT) en reuniones semanales, durante las cuales (mediante un proceso iterativo) fueron identificándose conceptos e ideas a partir del análisis de las piezas, construyendo conjuntamente las categorías y subcategorías emergentes, y confeccionando el informe narrativo final. El tamaño definitivo de la muestra estuvo sujeto al criterio de saturación teórica, y fue alcanzado con las 30 piezas seleccionadas inicialmente. 

### Variables

*Para el análisis cuantitativo*

Se analizó si cada pieza incluía recomendaciones sobre prevención primaria y secundaria de cáncer mamario, documentando los métodos de tamizaje recomendados, la edad de inicio y finalización y la periodicidad propuesta, y evaluando además la concordancia de dicha recomendación con las del INC. Adicionalmente, se analizó si eran mencionados beneficios y riesgos del tamizaje. 

*Para el análisis cualitativo*

Se analizaron cinco aspectos principales: la estructura y contenido de las piezas de difusión, la función de la imagen dentro de la comunicación visual, el diseño de la campaña, los recursos connotativos empleados, y la relación que se establece entre la pieza y el receptor (material suplementario).

### Análisis de los datos

*Análisis cuantitativo*

El análisis de la concordancia de los datos obtenidos con las recomendaciones del INC fue expresado como proporciones.

### Aspectos éticos

Este estudio fue aprobado (aprobación 4039) por el Comité de Ética de Protocolos de Investigación del Hospital Italiano de Buenos Aires (IRB00010193).

## RESULTADOS

De las 74 instituciones cuyos perfiles de redes sociales fueron revisados, 54 (73%) publicaron al menos una pieza de difusión sobre cáncer mamario durante octubre del 2019 (Figura 1), con un total de 171 piezas que cumplieron con los criterios de inclusión.

**Figura 1:** Diagrama de flujo que esquematiza el proceso de búsqueda e identificación de piezas de difusión vinculadas al cáncer mamario publicadas durante octubre de 2019 en los sitios oficiales de las 74 instituciones de salud de Argentina incluidas en nuestra revisión.

### Aspecto cuantitativo

Se encontró que 46 de las 54 instituciones (85%) realizaron una recomendación sobre prevención secundaria. De estas 46, 32 (69,5%) incluyeron a la mamografía, sola o acompañada de otras estrategias, como el examen físico mamario realizado por un profesional, que también fue recomendado por cinco instituciones (10,8%) (Tabla 2).

**Tabla 2:** Estrategias sugeridas por las 46 instituciones que realizaron recomendaciones sobre prevención secundaria en la revisión de las campañas de difusión de la temática del cáncer de mama durante octubre de 2019 en Argentina.

Tipo de institución	n	Recomendado por el INC		No recomendado por el INC		
		Mamografía (%)	Examen físico de las mamas (%)	Autoexamen mamario (%)	Ecografía (%)	Otros ^a^ (%)
Sistema de salud en Argentina						
Ministerios de salud	22	18 (81,8)	4 (18,1)	7 (31,8)	1 (5,5)	11 (50,0)
Sector del seguro social obligatorio	13	9 (69,2)	1 (7,7)	4 (30,7)	1 (7,7)	8 (61,5)
Empresas de medicina prepaga	8	2 (25,0)	0 (0,0)	6 (75,0)	1 (12,5)	6 (75,0)
Sociedades científicas	2	2 (100,0)	0 (0,0)	1 (50,0)	1 (50,0)	2 (100,0)
Organizaciones de la sociedad civil	1	1 (100,0)	0 (0,0)	0 (0,0)	0 (0,0)	1 (100,0)
Total	46	32 (69,5)	5 (10,8)	18 (39,1)	4 (8,7)	28 (60,8)

Una misma institución puede haber recomendado más de una estrategia como prevención secundaria de cáncer de mama, por eso la suma de los porcentajes puede exceder el 100%.

a Otros: consultar al médico de cabecera (n=12), ginecólogo (n=4), mastólogo (n=2), y realizar «controles médicos» sin especificar (n=13).

Entre las recomendaciones no concordantes con el INC, realizar autoexamen mamario fue mencionada por 18 instituciones (39%), cinco de ellas con una periodicidad mensual. En ocho instituciones (17%) fue la única estrategia de tamizaje recomendada, mientras que la ecografía mamaria fue recomendada por cuatro instituciones (8,7%).

Como se muestra en la Tabla 3, de las 32 instituciones que recomendaron la realización de mamografía, un total de 22 (69%) especificaron la edad de inicio y solo 9 (28%) la de finalización del tamizaje, y 20 (63%) de ellas explicitaron la periodicidad recomendada. 

El Ministerio de Salud de la Nación fue la única institución que emitió una recomendación totalmente concordante con la del INC.

**Tabla 3:** Recomendaciones sobre las edades de inicio, finalización, y periodicidad del tamizaje con mamografía comunicadas por 32 instituciones publicadas durante octubre de 2019 en las redes sociales oficiales de instituciones de Argentina.

Tipo de institución	n	Recomendación por edades							
		Inicio (años)			Finalización (años)		Periodicidad		
		NA	35-45	50 ^a^	NA	65-70 ^a^	NA	Anual	Bienal ^a^
Sistema de salud en Argentina									
Ministerios de salud	18	5 (27,8)	6 (33,3)	7 (38,9)	12 (66,7)	6 (33,3)	8 (44,4)	7 (38,9)	3 (16,7)
Sector del seguro social obligatorio	9	4 (44,5)	3 (33,3)	2 (22,2)	7 (77,8)	2 (22,2)	3 (33,3)	4 (44,5)	2 (22,2)
Empresas de medicina prepaga	2	1 (50,0)	1 (50,0)	0 (0,0)	2 (100,0)	0 (0,0)	1 (50,0)	1 (50,0)	0 (0,0)
Sociedades científicas	2	0 (0,0)	2 (100,0)	0 (0,0)	2 (100,0)	0 (0,0)	0 (0,0)	2 (100,0)	0 (0,0)
Organizaciones de la sociedad civil	1	0 (0,0)	1 (100,0)	0 (0,0)	0 (0,0)	1 (100,0)	0 (0,0)	1 (100,0)	0 (0,0)
Total	32	10 (31,2)	13 (40,7)	9 (28,1)	23 (71,9)	9 (28,1)	12 (37,5)	15 (46,9)	5 (15,6)

NA: no aclarado.

a Recomendaciones concordantes con el Instituto Nacional del Cáncer.

De las 54 instituciones analizadas, 44 (81%) mencionaron beneficios de realizar tamizaje y solamente tres los expresaron en forma cuantitativa (dos de ellas en términos relativos y una, en términos absolutos). Entre los beneficios clínicamente relevantes, el aumento de las probabilidades de curación fue el más mencionado (42%) seguido por la disminución de la mortalidad (18%), el aumento de la supervivencia (11 %) y el tratamiento precoz o menos invasivo (14,8%). Por el contrario, seis instituciones (11%) mencionaron a la «prevención» como un beneficio del tamizaje y 19 (35%) al diagnóstico precoz como un beneficio en sí mismo (Tabla 4). Ninguna institución mencionó posibles daños del tamizaje.

**Tabla 4:** Beneficios argumentados para respaldar la conveniencia de realizar tamizaje de cáncer mamario mediante las estrategias propuestas por 54 instituciones de Argentina durante octubre de 2019

Tipo de institución	n	Aumento de la probabilidad de curación (%)	Reducción de la mortalidad (%)	Aumento de la supervivencia (%)	Tratamiento precoz / menos invasivo (%)	Detección precoz como beneficio *per se* (%)	Prevención como fin en sí mismo (%)
Sistema de Salud en Argentina							
Ministerios de salud	25	10 (40,0)	4 (16,0)	0 (0,0)	3 (12,0)	9 (36,0)	3 (12,0)
Sector del seguro social obligatorio	15	5 (33,3)	2 (13,3)	2 (13,2)	2 (13,3)	5 (33,3)	3 (20,0)
Empresas de Medicina Prepaga	10	5 (50,0)	2 (20,0)	2 (20,0)	2 (20,0)	2 (20,0)	0 (0,0)
Sociedades científicas	3	3 (100,0)	1 (33,3)	2 (66,6)	1 (33,3)	2 (66,6)	0 (0,0)
Organizaciones de la Sociedad Civil	1	0 (0,0)	1 (100,0)	0 (0,0)	0 (0,0)	1 (100,0)	0 (0,0)
Total	54	23 (42,6)	10 (18,5)	6 (11,1)	8 (14,8)	19 (35,1)	6 (11,1)

Una misma institución puede haber argumentado más de un beneficio de realizar tamizaje de cáncer de mama, por eso la suma de porcentajes puede exceder el 100%.

Las piezas de 6 de las 54 instituciones (11%) mencionaron un aval o entidad citada como referencia y/o fuente de información de la recomendación emitida, que en todos los casos no fueron concordantes con las del INC (datos no mostrados).

### Aspecto cualitativo

Fueron analizadas 30 piezas de difusión (material suplementario) e identificadas las principales categorías emergentes (y sus subcategorías), que se describen a continuación. 

*a. Las imágenes y el diseño de las piezas*

Las imágenes fueron clasificadas según la función que cumplen dentro de la pieza y el contenido del mensaje que transmiten. 

*Función de la imagen dentro de la pieza de difusión*

Para atraer la atención, una estrategia muy utilizada es la exposición de cuerpos femeninos desnudos piezas 1 y 2 del material suplementario (MS) Por su parte, aquellas que buscan crear identificación con el receptor lo hacen mostrando mujeres con cuerpos estereotipados según el «ideal» hegemónico de belleza actual (piezas 1 y 3 del MS), o también representando a la mujer «luchadora», utilizando imágenes de pañuelos, símbolo de empoderamiento y de la reivindicación de los derechos de las mujeres (pieza 5 del MS). Además, las imágenes sirven de apoyo de la información que brinda el texto (piezas 4 y 6 del MS), y en ocasiones solo tienen un fin estético, (piezas 8 y 9 del MS) mostrando cuerpos que recuerdan a obras de arte (piezas 10, 11 y 12 del MS).

*Contenido del mensaje transmitido por la imagen (estereotipos de género y belleza)*

Predominan las imágenes de mujeres delgadas y jóvenes, fuera de la edad recomendada de tamizaje (piezas 1, 14 y 16 del MS), y con características que responden al estereotipo de belleza socialmente más aceptado actualmente (pieza 13 del MS). Se destaca una única pieza que muestra diversidad en las mujeres representadas (pieza 4 del MS). Las mujeres impresionan estar sanas, sin que se muestren mujeres bajo tratamientos oncológicos o transitando estadios avanzados de la enfermedad (pieza 13 del MS). En muchas piezas se ven mujeres sin rostro, centrándose la atención en sus manos, el torso (muchas veces desnudo), o solo en una mama (piezas 12, 14 y 15 del MS), y mostrándose muchas veces poses que le otorgan a la imagen una connotación erótica (pieza 1 del MS).

Prevalecen conceptos que refuerzan el estereotipo del género femenino, predomina el color rosado, las letras cursivas, y el uso de corazones o flores (piezas 17, 18 y 30), y se muestra a la mujer con una actitud pasiva, sonriente y delicada (piezas 16,19 y 20). 

*b. El texto*

Los elementos textuales que componen las piezas de difusión cumplen diversas funciones: concientización, persuasión y transmisión de información. 

*Concientización y persuasión*

Los elementos textuales apuntan a consolidar el concepto de octubre como mes dedicado al cáncer mamario (piezas 19, 20 y 21 del MS). Se observa que suelen aplicarse estrategias de persuasión interpelando a la receptora «¿Cuándo fue tu último chequeo?» y afirmando «La prevención está en tus manos» (piezas 5, 11 y 22 del MS). Algunas piezas incluyen signos de alarma o falsas creencias en torno al cáncer mamario (piezas 4 y 23 del MS). 

*(Des) Información transmitida*

Prevalece la ausencia de segmentación del mensaje por edades, la promoción de prácticas sin respaldo científico, la sobrestimación de sus beneficios y la omisión de los riesgos derivados de su implementación. Los mensajes suelen estar dirigidos a mujeres de todas las edades, la pregunta «¿ya te realizaste los estudios anuales?» parece transmitir que el tamizaje se debe realizar a cualquier edad sin distinción (pieza 21 del MS).

Un gran número de las piezas resaltan la importancia de la prevención con frases de alto impacto como «La prevención salva vidas» (piezas 10, 24 y 25 del MS), alimentando la falsa idea de que se podría evitar el desarrollo de todo cáncer, realizando estudios oportunamente. Por otro lado, muchas campañas recomiendan el autoexamen mamario, sin contar con evidencia que lo avale, ya que no se encontró que su implementación disminuya la mortalidad ^19^. Aun así, eslóganes como «Hoy tu mano salva vidas» y «Tócalas una vez al mes», brindan consejería sin respaldo científico (piezas 3 y 8 del MS).

Se observó una tendencia a sobreestimar los beneficios del tamizaje mediante afirmaciones como «Un estudio a tiempo puede salvarte la vida» (pieza 1 del MS), sin mencionar en qué magnitud el tamizaje reduce la mortalidad. Por su parte, en los enunciados como «A no bajar los brazos, se puede» (pieza 25 del MS) prevalece la idea de que el cáncer siempre se puede curar o que su evolución está condicionada por las conductas adoptadas. 

*c. Las imágenes, el diseño de la pieza y el texto: sentimientos y emociones evocados por las piezas de difusión*

Tanto las imágenes como la información que transmiten los elementos textuales pueden evocar diversos sentimientos y emociones en el público receptor. 

Muchas campañas evocan compromiso y unidad. Frases como «Por mí, por vos, por todas», «Hoy nos unimos contra el cáncer de mama», transmiten que la «lucha» es colectiva, como un fenómeno de responsabilidad social (piezas 4, 7 y 8 del MS).

Por otro lado, hay una tendencia a responsabilizar a la mujer afirmando «La prevención está en tus manos», (pieza 1 del MS) que al asegurar que el cáncer se puede prevenir y que hacerlo depende de la propia mujer, puede generar culpa y estigmatización en aquellas que no hayan realizado los estudios recomendados o que hayan recibido un diagnóstico de cáncer.

Algunas piezas evocan preocupación y miedo al resaltar «1 de cada 8 mujeres puede sufrir cáncer de mama, a mayor edad, mayor riesgo» (pieza 27 del MS), mientras otras piezas transmiten esperanza «Un estudio a tiempo puede salvarte la vida» (pieza 27 del MS). Es frecuente que ambos mensajes coexistan en la misma campaña, primero afirmando que el riesgo es «alto» y luego, recomendando estudios para «evitarlo» (pieza 7 del MS).

Prevalece la importancia del empoderamiento, definido por estas campañas como forma de responsabilizarse por la propia salud, y de realizar los estudios indicados (pieza 5).

Por su parte, se hace referencia al heroísmo en aquellas mujeres que logran curarse y se recurre al lenguaje épico. Se habla de la «Lucha», «Vencer esta enfermedad» (piezas 7, 24 y 26 del MS), lo cual da a entender que los resultados dependen de la fuerza y la voluntad individual y no considera otro tipo de variables, como las características propias del tipo de cáncer ^20^ o los determinantes sociales de la paciente.

Muchas piezas también transmiten una noción de obligatoriedad «¿Ya te realizaste los estudios anuales?», «A no bajar los brazos», dando por sentado que existe una única opción válida: realizar estudios y luego tratamiento. También se observa que las recomendaciones suelen estar formuladas en modo imperativo: «Realizá la primera mamografía a los 35 años» (piezas 3, 16 y 25 del MS).

## DISCUSIÓN

Luego de analizar cuantitativamente las campañas de cáncer de mama publicadas en Argentina en octubre del 2019, se observó que predomina la emisión de mensajes no concordantes con el INC, el consejo de realizar la mamografía a edades más tempranas y/o con intervalos más frecuentes, y/o a omitir la edad de finalización o a realizar autoexamen mamario. Si bien es deseable que la mujer detecte señales de alerta, el autoexamen no debe considerarse una estrategia de tamizaje ya que puede llevar a falsos reaseguros ^19^.

A su vez, las campañas solo comunicaron los potenciales beneficios del tamizaje sin mencionar potenciales daños. En las pocas piezas que cuantificaron los beneficios, lo hicieron utilizando medidas de efecto en términos relativos, cuando lo recomendable es exponerlos en términos absolutos ya que transmiten una idea más clara del potencial beneficio de realizar una mamografía ^21^.

Este sesgo en las campañas a favor de la realización del tamizaje desoye la evidencia creciente que cuestiona su real efectividad y alerta respecto de sus posibles daños ^10^. Asimismo, genera falsas expectativas ya que asume que la enfermedad tiene un comportamiento lineal y predecible, tanto prevenible como curable mediante las estrategias recomendadas, lo que constituye una interpretación falsamente simplificada de su comportamiento biológico, y oculta el amplio espectro de su potencial evolución ^20^ ^,^ ^22^.

Por último, resulta llamativo que no sea citada como fuente una entidad nacional y oficial como el INC, que cuenta con recomendaciones sólidamente documentadas sobre el cáncer mamario.

Desde lo cualitativo, se destaca la profundización de estereotipos de género y belleza y la imposición de un camino hegemónico como el único y válido para transitar el proceso salud-enfermedad.

El concepto de feminidad que prevalece parte de un sistema de género binario y heteronormativo en el cual a la mujer se la vincula con estereotipos como el color rosado, lo delicado y lo pasivo ^23^ ^,^ ^24^, que podrían actuar como barreras a la llegada de la información y al acceso a cuidados de la salud de colectivos que no se sientan representados por estos mensajes.

Asimismo, las imágenes de cuerpos desnudos y en poses provocativas perpetúan la sexualización y cosificación de la mujer ^25^ y se ven reforzados ciertos cánones de belleza en los que se impone como valores a la delgadez y a la juventud ^26^, hallazgos coincidentes con investigaciones realizadas en otros países ^26^ ^,^ ^27^.

En tercer lugar, prevalece en las campañas analizadas la imposición de una única opción válida de transitar el proceso salud-enfermedad, que transcurre a través de la realización de pruebas de tamizaje y eventuales tratamientos.

Los mensajes analizados reducen este proceso a la responsabilidad individual, excluyen los factores económicos, políticos y culturales ^28^, culpabilizan y estigmatizan a aquellas mujeres que deciden no realizar tamizaje, no logran acceder a él o, incluso, a quienes reciben un diagnóstico de cáncer ^20^. Se emite un mensaje de «empoderamiento femenino», pero desde una mirada normativa que apela al miedo y a la culpa como herramientas de persuasión, y basada en el modelo médico-hegemónico ^29^ ^,^ ^30^.

Asimismo, se enaltece la imagen de la mujer que decide «luchar contra el cáncer» mediante metáforas de guerra y la comparación de la enfermedad con una batalla que convierte a aquella mujer que se ha curado en «vencedora», lo cual implica que depende de la actitud y estilo de afrontamiento de la mujer ^30^. Se crea así la imagen de mujer fuerte, empoderada, luchadora, (*she-ro* del inglés *she is a hero*: ella es una heroína) ^24^. La contracara la representan las mujeres que se encuentran atravesando la enfermedad, o que fallecen a causa de ella, quienes se encuentran invisibilizadas o que, incluso, con el uso de estas metáforas se deja entrever que han perdido o «fracasado», situación también documentada en España ^20^.

Entre las limitaciones de este trabajo se menciona que el universo definido como recorte de esta investigación se limitó al análisis de las piezas de difusión emitidas por instituciones vinculadas con el cuidado de la salud, sin evaluar los mensajes emitidos por otro tipo de líderes de opinión como son las personas famosas (por ejemplo, del ambiente artístico o deportivo), cuyas opiniones y relatos vitales pueden ejercer influencias muy potentes en quienes los reciben.

Si bien existen antecedentes bibliográficos vinculados con esta temática ^13^ ^,^ ^31^, no se han encontrado otros estudios que hayan analizado la información transmitida por campañas de difusión sobre el cáncer mamario en Argentina. Consideramos que esta investigación es el primer paso para plantear un lugar clave donde poner los esfuerzos para informar de manera precisa a las mujeres, con información clara y restringida a recomendaciones que cuenten con aval científico de alta calidad.

La información inexacta e incompleta obstaculiza que las mujeres puedan realizar una correcta evaluación de sus riesgos y beneficios, y decidir de manera libre e informada respecto de someterse o no a una prueba de tamizaje. Consideramos fundamental avanzar hacia un modelo de toma de decisiones compartidas en el que se involucre a las mujeres en la decisión de realizar o no una intervención médica y se consideren sus valores y preferencias ^32^. Esta forma de trabajo empodera a la paciente, quien toma un rol activo y responsable de sus decisiones en salud, con expectativas realistas y, por ende, mejora las probabilidades de lograr los resultados más deseables ^33^.

En conclusión, los mensajes emitidos sobre tamizaje de cáncer mamario en octubre de 2019 por las instituciones incluidas fueron en su mayoría no concordantes con el INC. Esto ocurrió a expensas de mensajes que recomiendan realizar mamografías a edades más tempranas, a intervalos más frecuentes, y la implementación de estrategias que no demostraron disminuir la mortalidad, como el autoexamen mamario. Asimismo, se observó una tendencia a sobrestimar los beneficios del tamizaje y en todos los casos se omitió mencionar potenciales daños asociados a su implementación.

Por otro lado, los mensajes analizados presentan al cáncer de mama como una enfermedad lineal y homogénea cuyo curso natural podría ser fuertemente modificado por acciones individuales, lo que constituye una interpretación falsamente simplificada, y genera confusión y falsas expectativas.

Por último, se observa que en estas campañas se intenta abordar una problemática, pero se acentúan otras, como la perpetuación de los estereotipos de género y belleza, el modelo médico-hegemónico y la culpabilización y estigmatización de quien no sigue la norma impuesta.

Consideramos deseable repensar el enfoque que presentan las campañas relacionadas al cáncer mamario. Proponemos que se trate de espacios donde se transmita información completa y balanceada que transparente posibles beneficios y riesgos de realizar o no tamizaje de acuerdo a la mejor evidencia disponible, así como que se favorezca la diversidad y se ponga fin a la perpetuación de estereotipos de género y belleza.
